# No association between genetic markers and hypertension control in multiple cross-sectional studies

**DOI:** 10.1038/s41598-023-39103-8

**Published:** 2023-07-21

**Authors:** Valeriya Chekanova, Julien Vaucher, Pedro Marques-Vidal

**Affiliations:** 1 National Medical Research Center of Cardiology, Moscow, Russia; 2 Department of Medicine, Internal Medicine, Lausanne University Hospital (CHUV) and University of Lausanne, Office BH10-642, Rue du Bugnon 46, 1011 Lausanne, Switzerland

**Keywords:** Cardiology, Risk factors, Disease prevention, Public health

## Abstract

We aimed to assess whether genetic markers are associated with hypertension control using two cross-sectional surveys conducted in Lausanne, Switzerland. Management of hypertension was assessed as per ESC guidelines using the 140/90 or the 130/80 mm Hg thresholds. One genetic risk score (GRS) for hypertension (18 SNPs) and 133 individual SNPs related to response to specific antihypertensive drugs were tested. We included 1073 (first) and 1157 (second survey) participants treated for hypertension. The prevalence of controlled participants using the 140/90 threshold was 58.8% and 63.6% in the first and second follow-up, respectively. On multivariable analysis, only older age was consistently and negatively associated with hypertension control. No consistent associations were found between GRS and hypertension control (140/90 threshold) for both surveys: Odds ratio and (95% confidence interval) for the highest vs. the lowest quartile of the GRS: 1.06 (0.71–1.58) p = 0.788, and 1.11 (0.71–1.72) p = 0.657, in the first and second survey, respectively. Similar findings were obtained using the 130/80 threshold: 1.23 (0.79–1.90) p = 0.360 and 1.09 (0.69–1.73) p = 0.717, in the first and second survey, respectively. No association between individual SNPs and hypertension control was found. We conclude that control of hypertension is poor in Switzerland. No association between GRS or SNPs and hypertension control was found.

## Introduction

Hypertension, a major cardiovascular risk factor, is the leading cause of premature morbidity and disability-adjusted life years worldwide, and a primary risk factor for coronary artery disease, stroke, heart failure, chronic kidney disease and dementia^1^. Several randomized controlled trials have shown that reduction of blood pressure levels reduces fatal and non-fatal CVD events^2,3^. Those findings prompted international societies to issue guidelines for the adequate management of hypertension^4,5^. Still, one-half to one fifth of patients treated for hypertension fail to reach target levels^6–10^. In Switzerland, one in five men and one in six women present with hypertension^10^, and control of hypertension is far from optimal, as only half of treated patients achieve adequate blood pressure levels^11^. Increased age, lower educational level or being male are associated with lower control rates^9^.

In the last years, several genetic variants related to hypertension^12^ have been identified, leading to the constitution of genetic risk scores (GRS)^13^ or polygenic risk scores (PRS)^14^ associated with the risk of developing the disease. A list of SNPs associated with treatment-resistant hypertension has also been published^15^, and some genetic variants have been suggested to interact with specific antihypertensive drugs. For instance, a genetic variant in the catechol-*O*-methyl transferase (*COMT*) gene was significantly associated with a lower systolic blood pressure (SBP) level among subjects treated with calcium channel blockers^16^, while SNP rs2106809 of the *ACE2* gene was associated with response to ACE inhibitors in women^17^. Indeed, it has been suggested that genotyping might improve hypertension management^18^, but in a previous study we failed to find any association between a GRS made of 362 SNPs and hypertension management^19^. Still, whether GRS or specific genetic variants might influence hypertension control has been little studied.

Hence, our study aimed to identify the prevalence and the possible effect of genetic markers in poor control of hypertension in the Swiss population.

## Methods

### Study population

The CoLaus|PsyCoLaus (www.colaus-psycolaus.ch) is a prospective cohort study established in 2003 following every 5 years a sample of the inhabitants of the city of Lausanne (Switzerland), aged 35–75 years at baseline^20^. In each survey, participants answered questionnaires, underwent a clinical examination, and blood samples were drawn for analyses. As information regarding type of antihypertensive drug treatment was incomplete in the baseline survey, data from the first (2009–2012) and the second (2014–2017) surveys were used.

### Blood pressure, hypertension definition and hypertension control

Blood pressure (BP) was measured using an Omron^®^ HEM-907 automated oscillometric sphygmomanometer after at least a 10-min rest in a seated position, and the average of the last two measurements was used^20^. Participants reporting being treated for hypertension were considered as controlled using two thresholds: if their SBP was < 140 mm Hg and their DBP was < 90 m mHg (140/90), or if their SBP was < 130 mm Hg and their DBP was < 80 mm Hg (130/80) and as uncontrolled otherwise^5,21^.

### Genetic analysis and genetic score

Genome-wide genotyping was performed using the Affymetrix 500 K SNP array. Subjects were excluded from the analysis in case of inconsistency between sex and genetic data, a genotype call rate of less than 90%, or inconsistencies of genotyping results in duplicate samples^20^. Quality control for SNPs was performed using the following criteria: monomorphic (or with minor allele frequency (MAF) < 1%), call rates less than 90%, deviation from the Hardy–Weinberg equilibrium (HWE) (p < 1 × 10^–6^). Phased haplotypes were generated using SHAPEIT2^22^. Imputation was performed using minimac3 and the Haplotype Reference Consortium version r1.1.

A genetic risk score (GRS) related to treatment-resistant hypertension consisting of 20 SNPs^15^, 18 of which were available in our database (Supplementary Table 1) was selected. The GRS was computed as a weighted sum of the different SNPs and values range between 0 and 17. Further, 133 individual SNPs related to response to specific antihypertensive drugs were included in the analysis (Supplementary Table 2).

### Antihypertensive drug treatment

Participants were asked if they received any drug treatment for hypertension. All drugs taken by the participants (including or excluding non-prescribed, over-the-counter drugs) were collected and coded according to the Anatomical Therapeutic Chemical (ATC) classification of the WHO. The following classes of antihypertensive drugs were considered (* = any code): C03* (diuretics); C07* (beta-blockers); C08* (calcium channel blockers); C09A* or C09B* (ACE inhibitors); C09C* or C09D* (angiotensin receptor blockers, ARBs) and C02*, C04* (other antihypertensives).

### Other covariates

Socio-demographic and lifestyle data were collected by questionnaire and included gender, age, educational level (low/middle/high), marital status (alone/couple), personal and family history of CVD, family history of hypertension, smoking (never/former/current) and alcohol consumption (yes/no). Total number drugs (including or excluding non-prescribed, over-the-counter [OTC] drugs) was considered as a proxy for the number of comorbidities, including hypertension.

Body weight and height were measured with participants barefoot and in light indoor clothes. Body weight was measured in kilograms to the nearest 100 g using a Seca^®^ scale (Hamburg, Germany). Height was measured to the nearest 5 mm using a Seca^®^ (Hamburg, Germany) height gauge^20^. Body mass index (BMI) was calculated and categorized into normal (< 25 kg/m^2^), overweight (25 ≤ BMI < 30 kg/m^2^) and obese (BMI ≥ 30 kg/m^2^).

### Inclusion and exclusion criteria

For the genetic analyses, only participants of Caucasian origin were considered eligible. Caucasian origin was defined as having both parents and grandparents born in a restricted list of countries (available from the authors)^20^. A detailed description of the genetic background of the CoLaus sample is provided elsewhere^23^.

Participants were included if they received any type of antihypertensive drug treatment. Participants were excluded if they lacked information regarding BP levels, genetic data or covariates.

### Statistical analysis

Statistical analyses were conducted using Stata v.16.1 (Stata Corp, College Station, TX, USA) separately for each survey. Results were expressed as number of participants (percentage) for categorical variables and as average (± standard deviation) for continuous variables. Bivariate comparisons between controlled and uncontrolled participants were performed using chi-square for categorical variables and Student’s t-test or Kruskal–Wallis nonparametric test for continuous variables. Multivariable analyses were conducted using logistic regression for categorical variables and results were expressed as multivariable-adjusted odds ratio (OR) and 95% confidence interval (CI). For multivariable analyses, two models were applied: models 1 and 2 used the GRS for resistant hypertension either as a continuous variable (model 1) or categorized in quartiles (model 2). Adjustment was performed on age (continuous), gender (women/men), education (high/middle/low), marital status (in couple/other), BMI categories (normal/ overweight/obese), smoking categories (never/former/current), alcohol consumption (yes/no), hypolipidemic drug treatment (yes/no), antidiabetic drug treatment (yes/no), parental history of hypertension (yes/no), sedentary behavior (yes/no) and number of drugs, including OTC.

The associations between individual SNPs and hypertension control were performed by comparing the distribution of the genotypes between controlled and uncontrolled participants taking specific antihypertensive drugs. Chi-square or Fisher’s exact test were conducted as appropriate.

Statistical significance was considered for a two-sided test with p < 0.05.

### Ethical statement

The institutional Ethics Committee of the University of Lausanne, which afterwards became the Ethics Commission of Canton Vaud (www.cer-vd.ch) approved the baseline CoLaus study. The approval was renewed for the first and the second follow-ups. The study was performed in agreement with the Helsinki declaration and its former amendments, and in accordance with the applicable Swiss legislation. All participants gave their signed informed consent before entering the study.

## Results

### Participants

Of the initial 5064 participants of the first follow-up, 2103 (41.5%) reported taking antihypertensive drugs and were considered as eligible. Of those, 955 (45.4%) were excluded due to lack of genetic data, 71 (3.4%) due to missing covariates, and 4 (0.2%) due to missing BP data. Of the initial 4881 participants in the second follow-up, 2330 (47.7%) reported taking antihypertensive drugs and were considered as eligible. Of those, 912 (39.0%) were excluded due to lack of genetic data, 110 (4.7%) due to missing covariates, and 151 (6.5%) due to missing BP data. Overall, 1073 and 1157 participants were included in the analyses from first and second surveys, respectively. Of the 1073 participants in the first follow-up, 693 (64.6%) also participated in the second follow-up, while 464 participants untreated for hypertension in the first follow-up were included in the second follow-up (40.1% of the sample).

The characteristics of the included and excluded participants are summarized in Supplementary Table 3. Included participants were older, of a lower educational level, had a higher BMI, were more frequently former smokers or drinkers, and more frequently treated for dyslipidemia and diabetes.

### *Control defined as SBP/DBP* < *140/90 mm Hg*

The number (prevalence) of controlled participants were 631 (58.8%) and 736 (63.6%) in first and second follow-ups, respectively. The results of the bivariate analysis of the factors associated with BP control for both follow-ups are provided in Table 1. In both follow-ups, controlled participants were significantly younger, received a higher number of drugs (including and excluding OTC) , while no difference was found for the GRS related to resistant hypertension. Controlled participants were more frequently women or current smokers in the first but not in the second follow-up **(**Table 1).

**Table 1:** Bivariate comparison between controlled and uncontrolled participants, first (2009–2012) and second (2014–2017) follow-ups of the CoLaus|PsyCoLaus study, Lausanne, Switzerland. Control defined as a systolic blood pressure < 140 mmHg and a diastolic blood pressure < 90 mmHg.

	First			Second		
	Uncontrolled	Controlled	p	Uncontrolled	Controlled	p
N	442	631		421	736	
Age (years)	66.9 ± 8.9	63.6 ± 9.4	< 0.001	70.0 ± 9.2	67.4 ± 9.5	< 0.001
Women (%)	200 (45.3)	321 (50.9)	0.070	201 (47.7)	379 (51.5)	0.220
Swiss national (%)	310 (70.1)	457 (72.4)	0.414	297 (70.6)	507 (68.9)	0.555
Education (%)			0.887			0.236
High	54 (12.2)	80 (12.7)		55 (13.1)	119 (16.2)	
Middle	100 (22.6)	149 (23.6)		95 (22.6)	177 (24.1)	
Low	288 (65.2)	402 (63.7)		271 (64.4)	440 (59.8)	
Married/couple (%)	260 (58.8)	340 (53.9)	0.109	238 (56.5)	409 (55.6)	0.751
Body mass index (kg/m^2^)	28.6 ± 5.0	28.2 ± 4.9	0.167	27.9 ± 4.9	28.0 ± 4.8	0.761
Body mass index categories (%)			0.400			0.866
Normal	99 (22.4)	164 (26.0)		117 (27.8)	195 (26.5)	
Overweight	198 (44.8)	272 (43.1)		183 (43.5)	321 (43,6)	
Obese	145 (32.8)	195 (30.9)		121 (28.7)	220 (29.9)	
Smoking categories (%)			0.001			0.340
Never	167 (37.8)	235 (37.2)		163 (38.7)	275 (37.4)	
Former	222 (50.2)	269 (42.6)		195 (46.3)	326 (44.3)	
Current	53 (12.0)	127 (20.1)		63 (15.0)	135 (18.3)	
Alcohol drinker (%)	339 (76.7)	444 (70.4)	0.022	270 (72.0)	461 (67.9)	0.166
Sedentary (%)	261 (71.5)	348 (69.6)	0.544	179 (62.8)	297 (61.6)	0.743
Treatment for (%)						
Dyslipidemia	189 (42.8)	306 (48.5)	0.064	168 (39.9)	329 (44.7)	0.113
Diabetes	69 (15.5)	102 (16.2)	0.807	59 (14.0)	116 (15.8)	0.425
Number of drugs, median [IQR]						
All, including OTC	4 [2–5]	4 [2–6]	0.075 §	4 [2–6]	5 [3–7]	< 0.001 §
All, excluding OTC	3 [2–5]	4 [2–6]	0.212 §	4 [2–6]	4 [2–6]	0.002 §
Antihypertensive drugs	1 [1, 2]	1 [1, 2]	0.848 §	1 [1, 2]	2 [1, 2]	0.065 §
Parental history of HT (%)	177 (40.1)	266 (42.2)	0.490	179 (42.5)	333 (45.2)	0.369
GRS for resistant hypertension	5.8 ± 2.7	5.9 ± 2.6	0.952	5.7 ± 2.6	5.7 ± 2.6	0.872

*HT* hypertension, *IQR* interquartile range, *OTC* over the counter, *SD* standard deviation. Results are expressed as number of participants (column %) for categorical variables and as average ± standard deviation or as median [interquartile range] for continuous variables. Between-groups comparisons performed using chi-square for categorical variables and student’s t-test or Kruskal–Wallis nonparametric test (§) for continuous variables.

The results of the multivariable analysis are provided in Table 2 for the first and the second follow-ups. In the first follow-up, increasing age, being married and presenting with obesity were associated with a lower likelihood of BP control, while total number of drugs (including OTC) was positively associated with BP control. No association was found for the GRS for resistant hypertension (Table 2). In the second follow-up, increasing age, being a man and having a lower educational level were associated with a lower likelihood of BP control, while hypolipidemic drug treatment was positively associated with BP control. No association was found with the GRS for resistant hypertension (Table 2).

**Table 2:** Multivariable analysis of the associations between clinical and genetic factors with blood pressure control, first (2009–2012) and second (2014–2017) follow-ups of the CoLaus|PsyCoLaus study, Lausanne, Switzerland. Control defined as a systolic blood pressure < 140 mmHg and a diastolic blood pressure < 90 mmHg.

	First follow-up (2009–2012)				Second follow-up (2014–2017)			
	Model 1	p	Model 2	p	Model 1	p	Model 2	p
Age (per 10 years)	0.59 (0.50–0.70)	< 0.001	0.60 (0.50–0.71)	< 0.001	0.61 (0.50–0.75)	< 0.001	0.61 (0.50–0.75)	< 0.001
Men vs. women	0.86 (0.62–1.18)	0.346	0.87 (0.63–1.19)	0.375	0.69 (0.48–0.97)	0.034	0.68 (0.48–0.96)	0.030
Swiss national vs. other	1.03 (0.74–1.43)	0.869	1.02 (0.74–1.41)	0.909	1.10 (0.77–1.58)	0.585	1.11 (0.78–1.58)	0.578
Education								
High	1 (ref.)		1 (ref.)		1 (ref.)		1 (ref.)	
Middle	0.98 (0.60–1.62)	0.946	0.96 (0.58–1.58)	0.877	0.82 (0.50–1.34)	0.421	0.81 (0.50–1.33)	0.408
Low	0.99 (0.63–1.55)	0.952	0.96 (0.62–1.50)	0.865	0.62 (0.39–0.98)	0.039	0.62 (0.39–0.97)	0.038
*P*-value for trend	0.952		0.865		0.039		0.038	
Married/couple vs. alone	0.74 (0.55–0.99)	0.043	0.73 (0.55–0.99)	0.040	1.02 (0.73–1.42)	0.920	0.99 (0.71–1.39)	0.976
Body mass index categories								
Normal	1 (ref.)		1 (ref.)		1 (ref.)		1 (ref.)	
Overweight	0.77 (0.54–1.10)	0.151	0.77 (0.54–1.10)	0.146	0.93 (0.63–1.36)	0.706	0.93 (0.63–1.35)	0.689
Obese	0.64 (0.43–0.95)	0.026	0.66 (0.44–0.97)	0.036	1.07 (0.69–1.67)	0.765	1.08 (0.69–1.68)	0.732
*P*-value for trend	0.026		0.036		0.765		0.732	
Smoking categories								
Never	1 (ref.)		1 (ref.)		1 (ref.)		1 (ref.)	
Former	0.90 (0.65–1.23)	0.498	0.89 (0.65–1.22)	0.484	0.92 (0.64–1.30)	0.627	0.91 (0.64–1.30)	0.616
Current	1.31 (0.84–2.05)	0.227	1.32 (0.85–2.05)	0.222	1.06 (0.66–1.71)	0.799	1.07 (0.67–1.72)	0.781
*P*-value for trend	0.227		0.222		0.799		0.781	
Alcohol drinker (yes vs. no)	0.74 (0.52–1.04)	0.082	0.74 (0.53–1.04)	0.083	1.02 (0.70–1.48)	0.915	1.04 (0.72–1.50)	0.831
Sedentary (yes vs. no)	0.93 (0.68–1.29)	0.681	0.94 (0.68–1.29)	0.701	1.08 (0.78–1.51)	0.637	1.08 (0.78–1.51)	0.645
Treatment for								
Dyslipidemia (yes vs. no)	1.38 (1.01–1.90)	0.046	1.34 (0.98–1.85)	0.068	1.40 (0.99–1.99)	0.059	1.39 (0.98–1.97)	0.062
Diabetes (yes vs. no)	1.20 (0.76–1.87)	0.435	1.18 (0.76–1.85)	0.462	0.95 (0.56–1.61)	0.846	0.97 (0.57–1.63)	0.902
Number of drugs, including OTC	1.07 (1.00–1.15)	0.056	1.07 (1.01–1.15)	0.046	1.05 (0.98–1.12)	0.206	1.05 (0.98–1.12)	0.193
Parental history of HT (yes vs. no)	1.03 (0.77–1.39)	0.830	1.03 (0.77–1.39)	0.822	0.92 (0.66–1.28)	0.610	0.91 (0.65–1.27)	0.583
Genetic risk score								
Resistant hypertension, continuous	–		1.01 (0.96–1.06)	0.768			1.02 (0.96–1.08)	0.535
Resistant hypertension, quartiles								
First	1 (ref.)		–		1 (ref.)		–	
Second	0.84 (0.56–1.24)	0.376	–		1.21 (0.79–1.85)	0.389	–	
Third	1.24 (0.83–1.86)	0.301	–		1.45 (0.93–2.28)	0.105	–	
Fourth	1.06 (0.71–1.58)	0.788	–		1.11 (0.71–1.72)	0.657	–	
*P*-value for trend	0.390				0.496			

*HT* hypertension, *OTC* over the counter, *ttt* treatment, – not included in the model. Results are expressed as odds ratio (95% confidence interval). Statistical analyses performed using logistic regression.

### *Control defined as SBP/DBP* < *130/80 mmHg*

Number (prevalence) of controlled participants was 337 (31.4%) and 393 (34.0%) in the first and second follow-ups, respectively. The results of the bivariate analysis of the factors associated with BP control for both follow-ups are provided in Supplementary Table 4. In both follow-ups, controlled participants were significantly younger, had a lower BMI, and received a higher number of drugs (including and excluding OTC) than uncontrolled participants. No differences were found regarding the GRS (Supplementary Table 4).

The results of the multivariable analysis are provided in Supplementary Table 5 for the first and the second follow-ups. In the first follow-up, increasing age, increasing BMI and alcohol consumption were associated with a lower likelihood of BP control, while antidiabetic drug treatment and total number of drugs (including OTC) was positively associated with BP control. No association was found with the GRS related to resistant hypertension (Supplementary Table 5). In the second follow-up, increasing age, increasing BMI and being a man were associated with a lower likelihood of BP control, while total number of drugs (including OTC) was positively associated with BP control. No association was found with the GRS related to resistant hypertension (Supplementary Table 5).

### Association between drug-specific SNPs and blood pressure control

The results of the associations between drug-specific SNPs and BP control according to the presence of the drug are summarized in Supplementary Fig. 1. Overall, very few significant (p < 0.05) associations were found, and only one SNP (rs675388 of *KCNJ1*) showed consistent associations with diuretic treatment in three of the four analyses performed.

## Discussion

Our results suggest that genetic markers are associated neither with hypertension control, nor with response to antihypertensive drugs in a sample of community-dwelling people.

### Characteristics of the participants

Included participants were older, more frequently male, and had a higher prevalence of other cardiovascular risk factors than excluded participants. This was expected, as our study focused on participants with hypertension, as hypertension rates increase with age and are frequently associated with other cormorbidities.

### Prevalence of controlled hypertension

Prevalence of controlled hypertension was below 60% when using the 140/90 threshold and decreased to less than one third when using the 130/80 threshold. Those values are close to those reported in Germany^24^, where 54% of participants treated for hypertension had a BP level below the 140/90 mm Hg. Those values are also comparable to a Swedish study^25^ where 59% of women and 48% of men treated for hypertension were controlled, or to a Greek study, which found a control rate among treated participants of 56% in women and 43% in men^26^. Conversely, our control rates were higher than reported in France (50%)^27^, an European study (47%)^28^ and a study conducted in the UK and Ireland (38%)^29^. Overall, our results suggest that management of hypertension in Switzerland is comparable or slightly better than other European countries. Nevertheless, control rates remain suboptimal, as at least four out of ten patients failed to achieve adequate BP levels.

### Factors associated with blood pressure control

Increasing age was negatively associated with hypertension control. Our findings are in agreement with studies conducted in Germany^30^ and Iran^31^ but not with another German^24^ or Swede^25^ studies, where no association was found. Possible explanations include the use of a higher threshold for BP control among the elderly^32,33^, or the avoidance of deleterious side effects due to low BP levels in elderly people by Swiss GPs. Still, recent data indicate that BP lowering in elderly people is safe and reduces CVD events^34^. Hence, BP lowering should be applied to elderly people to the same extent as to younger people, as stated in the current ESC guidelines^5^.

Increased BMI levels were negatively associated with hypertension control using the 130/80 threshold but less so using the 140/90 threshold. Our findings replicate those of a prospective study conducted in the UK, where hypertension prevalence increased and hypertension control decreased with increasing BMI^35^. Hypertension in obese patients is mainly due to increased cardiac output with “inadequately normal” peripheral resistance due to dysfunction of the renin–angiotensin–aldosterone system and the cardiac natriuretic peptide system^36^. This dysfunctional state could make BP control harder in obese people. Overall, our results indicate that obese people could benefit from stronger lifestyle and antihypertensive treatment than normal weight people.

Total number of drugs including OTCs, was positively associated with BP control. The increasing number of drugs could be related to an increased number of antihypertensive drugs^37^. Still, no association between number of antihypertensive drugs and hypertension control was found, a finding in agreement with a German^30^, but not with a Greek study^26^, where number of antihypertensive drugs was negatively associated with hypertension control. Interestingly, presence of hypolipidemic drug treatment was associated with a better control of hypertension, suggesting that participants with multiple risk factors might be more health-conscious or more closely monitored.

### Genetics and hypertension control

No association was found between the GRS and hypertension control in both surveys and for both thresholds. Our results are in line with a previous paper from our group where no association between a 362-SNP GRS and BP control was found^19^ and with a recent Finnish study, where no clear association between a 793-SNP PRS and BP control was found^38^. A likely explanation is that the effect of those GRS is too small to be detected with the current sample size. For instance, a genome-wide association study identified over 500 loci associated with BP traits^39^, but no BP score was derived, and together these loci only explained between 3.5%^40^ and 13%^41^ of the trait variance. Hence, the effect of GRS on BP levels might be too small to be clinically relevant in general practice. Another possibility is that antihypertensive drug treatment was stronger among participants with higher GRS. Still, no association was found between number of antihypertensive classes and the GRS (Supplementary Fig. 2).

Similarly, no significant association was found between individual SNPs and hypertension control according to antihypertensive drug used, the most consistent association being found between rs675388 of *KCNJ1* and diuretic treatment. Our findings do not replicate those of a previous review^42^, but are in line with a Finnish study, where higher PRS for hypertension tended to be associated with a lower response to diuretic treatment^38^ and to hypertension onset^14^. Overall, our results are in line with current recommendations^4,5^ and do not support the use of GRS or individual SNPs to manage hypertension.

### Importance for clinical practice

When managing patients with hypertension, doctors should focus on clinical factors such as age, increased BMI, and possibly gender and polypharmacy. The use of a GRS or individual SNPs to direct treatment is not recommended.

### Study limitations

This study has several limitations worth acknowledging. Firstly, the sample size was relatively small, and our study was likely underpowered to detect the minute associations between the GRS and hypertension control. Still, based on our findings, it is unlikely that the effects of the GRS, if any, could be of interest in clinical practice. Secondly, it was not possible to adequately collect the posology of the antihypertensive treatment. Hence, we could not determine if the participants were receiving the maximal dose. Thirdly, included participants presented with more comorbidities than excluded ones, which might have blurred the association between GRS and hypertension control. Hence, it would be important to replicate our study in a larger sample including participants with hypertension but devoid of other comorbidities. Finally, the SNPs used to compute the GRS for resistant hypertension were not independent, as indicated in Supplementary Table 7; hence, the weight of some genes on the GRS was overestimated. Still, restricting the GRS to one single SNP per gene (rs17035646 for *CASZ1* and rs77270397 for *EEF1DP3, FRY-AS1*) led to similar findings, i.e., the lack of association between the short GRS and hypertension control (Supplementary Tables 8 to 11).

## Conclusion

Control of hypertension is poor in Switzerland, namely among older adults and possibly among overweight or obese subjects. No association between GRS or individual SNPs and hypertension control could be found.

## Supplementary Information

Supplementary Figure 1.

Supplementary Figure 2.

Supplementary Tables.

## Data Availability

The CoLaus|PsyCoLaus cohort data used in this study cannot be fully shared as they contain potentially sensitive patient information. As discussed with the competent authority, the Research Ethic Committee of the Canton of Vaud, transferring or directly sharing this data would be a violation of the Swiss legislation aiming to protect the personal rights of participants. Non-identifiable, individual-level data are available for interested researchers, who meet the criteria for access to confidential data sharing, from the CoLaus Datacenter (CHUV, Lausanne, Switzerland). Instructions for gaining access to the CoLaus data used in this study are available at https://www.colaus-psycolaus.ch/professionals/how-to-collaborate/.
