# Supplementary material for: No association between genetic markers and hypertension control in multiple cross-sectional studies
Source: Sci Rep. 2023 Jul 21;13:11811. doi: 10.1038/s41598-023-39103-8 (PMC10362004; doi:10.1038/s41598-023-39103-8)
Supplement: Supplementary file 1 — Supplementary Figure 1. [file 41598_2023_39103_MOESM1_ESM.docx]

# Supplementary figure legends

**Supplementary figure 1**: Manhattan plots of the associations between drug-specific SNPs and blood pressure control, first (2009-2012) and second (2014-2017) follow-ups of the CoLaus|PsyCoLaus study, Lausanne, Switzerland.

Panels A and B: control defined as a systolic blood pressure <140 mm Hg and a diastolic blood pressure <90 mm Hg. The upper graph (panel A) corresponds to the first follow-up, the lower one (panel B) to the second follow-up. The horizontal dotted line corresponds to a p-value of 0.05. Black, ACE inhibitors; orange, angiotensin-receptor blockers; green, beta-blockers; yellow, calcium channel blockers, and blue, diuretics.

Panels C and D: control defined as a systolic blood pressure <130 mm Hg and a diastolic blood pressure <80 mm Hg. The upper graph corresponds to the first follow-up, the lower one to the second follow-up. The horizontal dotted line corresponds to a p-value of 0.05. Black, ACE inhibitors; orange, angiotensin-receptor blockers; green, beta-blockers; yellow, calcium channel blockers, and blue, diuretics.
