# Supplementary material for: No association between genetic markers and hypertension control in multiple cross-sectional studies
Source: Sci Rep. 2023 Jul 21;13:11811. doi: 10.1038/s41598-023-39103-8 (PMC10362004; doi:10.1038/s41598-023-39103-8)
Supplement: Supplementary file 2 — Supplementary Figure 2. [file 41598_2023_39103_MOESM2_ESM.docx]

**Supplementary figure 2**: association between the genetic risk score (GRS) for resistant hypertension and number of antihypertensive drugs, first (2009-2012) and second (2014-2017) follow-ups of the CoLaus|PsyCoLaus study, Lausanne, Switzerland. GRS, genetic risk score.
