# Supplementary material for: No association between genetic markers and hypertension control in multiple cross-sectional studies
Source: Sci Rep. 2023 Jul 21;13:11811. doi: 10.1038/s41598-023-39103-8 (PMC10362004; doi:10.1038/s41598-023-39103-8)
Supplement: Supplementary file 3 — Supplementary Tables. [file 41598_2023_39103_MOESM3_ESM.docx]

**Supplementary tables**

**Supplementary table 1**: components of the genetic risk scores for treatment-resistant hypertension and hypertension.

| **Condition / SNPs** | **Gene** | **Major or minor allele** | **Weight** |
| --- | --- | --- | --- |
| **Treatment-resistant HT** |  |  |  |
| rs138399316 | BPHL | NA |  |
| rs12046278 | CASZ1 | major | 0.71 |
| rs17035646 | CASZ1 | minor | 1.36 |
| rs34071855 | CASZ1 | major | 0.72 |
| rs880315 | CASZ1 | major | 0.74 |
| rs114349263 | CDH18 | minor | 0.08 |
| rs185169399 | CDH18 | major § | 11.96 |
| rs74725390 | COBL, POM121L12 | minor | 1.70 |
| rs11674660 | DNMT3A, DTNB | minor | 1.53 |
| rs12050053 | EEF1DP3, FRY-AS1 | minor | 2.43 |
| rs77270397 | EEF1DP3, FRY-AS1 | minor | 2.09 |
| rs12665245 | ENPP3 | major | 0.36 |
| rs146183009 | ICMT | minor | 2.49 |
| rs111285947 | LINC00670 | minor | 3.89 |
| rs143255889 | LINC01519 | minor | 3.10 |
| rs1651805 | LSM14A, KIAA0355 | minor | 1.84 |
| rs111281682 | MYL10, CUX1 | major | 0.72 |
| rs76967376 | MYO5B | minor | 2.65 |
| rs4844662 | PLXNA2 | minor | 1.31 |
| rs114511751 | TMCC2 | minor | 2.44 |

HT, hypertension; NA, not available; SNP, single nucleotide polymorphism. § not used in calculations as all participants were homozygous. From reference (1)

**Supplementary table 2**: single nucleotide polymorphisms (SNPs) associated with specific antihypertensive drugs.

| **Drug/SNPs** | **Gene** | **Reference** |
| --- | --- | --- |
| **ACE inhibitors** |  |  |
| rs495828 | ABO | (2) |
| rs1799752 | ACE | (2) |
| rs4344 | ACE | (2) |
| rs4359 | ACE | (2) |
| rs2106809 | ACE1 | (2) |
| rs4961 | ADD1 | (2) |
| rs4762 | AGT | (2) |
| rs5051 | AGT | (2) |
| rs699 | AGT | (2) |
| rs7079 | AGT | (2, 3) |
| rs1403543 | AGTR1 | (3) |
| rs5182 | AGTR1 | (2) |
| rs5186 | AGTR1 | (2) |
| rs5194 | AGTR1 | (3) |
| rs1799722 | BDKRB2 | (2, 4) |
| rs8012552 | BDKRB3 | (2) |
| rs3025058 | MMP3 | (2) |
| rs2070744 | NOS3 | (2, 4) |
| rs3918188 | NOS3 | (5) |
| rs3918226 | NOS3 | (5) |
| rs5522 | NR3C2 | (2, 3) |
| rs2229437 | PRCP | (2) |
| rs11209716 | PTGER3 | (2) |
| rs4742610 | PTPRD | (2) |
| rs699947 | VEGFA | (2) |
| **Angiotensin receptor blockers** |  |  |
| rs5051 | AGT | (2) |
| rs699 | AGT | (2) |
| rs5186 | AGTR1 | (2) |
| rs1367117 | APOB | (2) |
| rs10737061 | CAMK1D | (6) |
| rs10752271 | CAMK1D | (6, 7) |
| rs10906202 | CAMK1D | (6) |
| rs4747995 | CAMK1D | (6) |
| rs1799998 | CYP11B2 | (2, 3) |
| rs1057910 | CYP2C9 | (2) |
| rs11020821 | FUT4 | (3) |
| rs3758785 | GPR83 | (3, 8) |
| rs3814995 | NPHS1 | (9, 10) |
| rs11649420 | SCNN1G | (3, 8) |
| rs6749447 | STK39 | (2) |
| **Beta-blockers** |  |  |
| rs3213619 | ABCB1 | (2) |
| rs1799752 | ACE | (2) |
| rs2106809 | ACE1 | (2) |
| rs2514036 | ACY3 | (6, 11) |
| rs2514037 | ACY3 | (6) |
| rs948445 | ACY3 | (6) |
| rs4961 | ADD1 | (2) |
| rs1801252 | ADRB1 | (6, 10, 12, 13) |
| rs1801253 | ADRB1 | (2, 6, 10, 12, 14-16) |
| rs5051 | AGT | (2) |
| rs699 | AGT | (2) |
| rs5186 | AGTR1 | (2) |
| rs261316 | ALDH1A2 | (17) |
| rs1051375 | CACNA1C | (2) |
| rs871606 | CHIC2 | (3) |
| rs294610 | FGD5 | (18) |
| rs1458038 | FGF5 | (3) |
| rs12595985 | FTO | (2) |
| rs9940629 | FTO | (2) |
| rs2144297 | GALNT2 | (2) |
| rs2144300 | GALNT2 | (2) |
| rs11064426 | GNB3 | (3) |
| rs2301339 | GNB3 | (3) |
| rs5443 | GNB3 | (3) |
| rs1024323 | GRK4 | (2, 3, 10, 12, 19) |
| rs1081058 | GRK4 | (3, 10, 12) |
| rs2960306 | GRK4 | (2, 3, 10, 19) |
| rs1799945 | HFE | (3) |
| rs688 | LDLR | (2) |
| rs11313667 | LRRC15 | (2, 10) |
| rs2932538 | MOV10 | (3) |
| rs292449 | NEDD4L | (2) |
| rs4149601 | NEDD4L | (2, 6, 20) |
| rs2070744 | NOS3 | (2) |
| rs11039149 | NR1H3 | (2) |
| rs1015710 | PLA2G4A | (2) |
| rs340874 | PROX1 | (2) |
| rs4648287 | PTGS2 | (2) |
| rs10739150 | PTPRD | (10) |
| rs12346562 | PTPRD | (2, 10) |
| rs201279313 | SLC25A31 | (2, 10, 21) |
| **Calcium channel blockers** |  |  |
| rs1045642 | ABCB1 | (22) |
| rs1799752 | ACE | (2) |
| rs2106809 | ACE1 | (2) |
| rs4961 | ADD1 | (2) |
| rs5186 | AGTR1 | (2) |
| rs564991 | APCDD1 | (3) |
| rs1051375 | CACNA1C | (2, 3, 23) |
| rs2238032 | CACNA1C | (2) |
| rs2239128 | CACNA1C | (2) |
| rr527974 | CACNA1C, CACNA1D | (24) |
| rs312481 | CACNA1C, CACNA1D | (24) |
| rs3774426 | CACNA1C, CACNA1D | (24) |
| rs2357928 | CACNB2 | (23) |
| rs5065 | CLCN6 | (2) |
| rs2246709 | CYP3A4 | (2, 25) |
| rs2740574 | CYP3A4 | (2, 25) |
| rs10364272 | CYP3A5 | (26) |
| rs11739136 | KCNMB1 and KCNIP1 | (2, 10, 27) |
| rs2301149 | KNCMB3 | (2, 10, 27) |
| rs4149601 | NEDD4L | (2) |
| rs10494366 | NOS1AP | (2) |
| rs2070744 | NOS3 | (2) |
| rs11039149 | NR1H3 | (2) |
| rs12221497 | NR1H3 | (2) |
| rs2279238 | NR1H3 | (2) |
| rs10898815 | NUMA1 | (3) |
| rs588076 | PICALM | (3) |
| rs12946454 | PLCD3 | (2, 3) |
| rs4742610 | PTPRD | (2) |
| rs1692743 | SIGLEC12, A1BG, F5 | (12) |
| rs4525 | SIGLEC12, A1BG, F5 | (12) |
| rs893184 | SIGLEC12, A1BG, F5 | (12) |
| rs3745009 | SLC14A2 | (2) |
| rs1123617 | SLC14A2 | (2) |
| rs2429427 | TANC2 | (2, 3) |
| **Diuretics** |  |  |
| rs11600347 | KCNJ1 | (2) |
| rs12795437 | KCNJ1 | (2) |
| rs59172778 | KCNJ1 | (2) |
| rs658903 | KCNJ1 | (2) |
| rs4132670 | [TCF7L2](https://www.ncbi.nlm.nih.gov/search/all/?term=human+TCF7L2&item=gene:6934:TCF7L2&prev-item=snp:4132670:rs4132670&theme=genetics) | (2) |
| rs7917983 | [TCF7L2](https://www.ncbi.nlm.nih.gov/search/all/?term=human+TCF7L2&item=gene:6934:TCF7L2&prev-item=snp:4132670:rs4132670&theme=genetics) | (2) |
| rs1799752 | ACE | (2, 28) |
| rs2106809 | ACE1 | (2) |
| rs4961 | ADD1 | (2, 29) |
| rs261316 | ALDH1A2 | (17) |
| rs5065 | CLCN6 | (2) |
| rs11993031 | CSMD1 | (6) |
| rs7387065 | CSMD1 | (6) |
| rs4551053 | EBF1 | (3) |
| rs1458038 | FGF5 | (3) |
| rs4784333 | FTO | (2) |
| rs2273359 | GNAS-EDN3 | (12) |
| rs5443 | GNB3 | (30) |
| rs675388 | KCNJ1 | (2) |
| rs6947309 | LUC7L2 | (2) |
| rs3025058 | MMP3 | (2) |
| rs4149601 | NEDD4L | (2, 6, 10, 12, 20, 31) |
| rs75982813 | NEDD4L | (2) |
| rs292449 | NEDD4L | (3) |
| rs4149601 | NEDD4L | (3) |
| rs2070744 | NOS3 | (2) |
| rs16960228 | PRKCA | (3, 10, 32) |
| rs4791040 | PRKCA | (2) |
| rs11240688 | REN | (2) |
| rs8085654 | ROCK1 | (6) |
| rs3184504 | SH2B3 | (3) |
| rs4506565 | TCF7L2 | (2) |
| rs12505746 | TET4 | (6) |
| rs2378479 | TLE1 | (2) |
| rs880054 | WNK1 | (2) |
| rs292449 | YEATS4 | (6, 31) |
| rs315135 | YEATS4 | (3, 6, 12, 33) |
| rs317689 | YEATS4 | (3, 6, 12, 33) |
| rs7297610 | YEATS4 | (2, 3, 6, 10, 12, 33) |

ACE, angiotensin-converting enzyme.

**Supplementary table 3**: characteristics of included and excluded participants, first (2009-2012) and second (2014-2017) follow-ups of the CoLaus|PsyCoLaus study, Lausanne, Switzerland.

|  |  | **First** |  |  | **Second** |  |
| --- | --- | --- | --- | --- | --- | --- |
|  | **Included** | **Excluded** | **p** | **Included** | **Excluded** | **p** |
| N | 1073 | 1030 |  | 1157 | 1173 |  |
| Age (years) | 65.0 ± 9.3 | 55.0 ± 10.0 | <0.001 | 68.3 ± 9.5 | 62.0 ± 10.8 | <0.001 |
| Women (%) | 521 (48.6) | 525 (51.0) | 0.268 | 580 (50.1) | 631 (53.8) | 0.077 |
| Swiss national (%) | 767 (71.5) | 467 (45.3) | <0.001 | 804 (69.5) | 573 (48.9) | <0.001 |
| Education (%) |  |  | <0.001 |  |  | <0.001 |
| High | 134 (12.5) | 266 (26.0) |  | 174 (15.0) | 275 (23.5) |  |
| Middle | 249 (23.2) | 277 (27.0) |  | 272 (23.5) | 294 (25.2) |  |
| Low | 690 (64.3) | 482 (47.0) |  | 711 (61.5) | 600 (51.3) |  |
| Married/couple (%) | 600 (55.9) | 598 (58.1) | 0.322 | 647 (55.9) | 569 (52.8) | 0.143 |
| Body mass index (kg/m^2^) | 28.4 ± 4.9 | 26.2 ± 4.6 | <0.001 | 28.0 ± 4.8 | 26.8 ± 4.7 | <0.001 |
| Body mass index categories (%) |  |  | <0.001 |  |  | <0.001 |
| Normal | 263 (24.5) | 440 (44.3) |  | 312 (27.0) | 364 (38.4) |  |
| Overweight | 470 (43.8) | 392 (39.5) |  | 504 (43.6) | 391 (41.2) |  |
| Obese | 340 (31.7) | 161 (16.2) |  | 341 (29.5) | 193 (20.4) |  |
| Smoking categories (%) |  |  | <0.001 |  |  | 0.001 |
| Never | 402 (37.5) | 411 (40.8) |  | 438 (37.9) | 430 (44.1) |  |
| Former | 491 (45.8) | 353 (35.1) |  | 521 (45.0) | 360 (36.9) |  |
| Current | 180 (16.8) | 243 (24.1) |  | 198 (17.1) | 186 (19.1) |  |
| Alcohol drinker (%) | 783 (73.0) | 705 (68.5) | 0.023 | 731 (69.4) | 631 (67.7) | 0.429 |
| Sedentary (%) | 609 (70.4) | 467 (58.0) | <0.001 | 476 (62.1) | 335 (54.2) | 0.003 |
| Treatment for (%) |  |  |  |  |  |  |
| Dyslipidemia | 495 (46.1) | 183 (17.8) | <0.001 | 497 (43.0) | 291 (24.8) | <0.001 |
| Diabetes | 171 (15.9) | 53 (5.2) | <0.001 | 175 (15.1) | 114 (9.7) | <0.001 |
| Number of drugs, median [IQR] |  |  |  |  |  |  |
| All, including OTC | 4 [2 - 6] | 1 [0 - 3] | <0.001 § | 4 [3 - 6] | 2 [1 - 5] | <0.001 § |
| All, excluding OTC | 4 [2 - 5] | 1 [0 - 3] | <0.001 § | 4 [2 - 6] | 2 [0 - 4] | <0.001 § |

OTC, over the counter; SD, standard deviation. Results are expressed as number of participants (column %) for categorical variables and as average ± standard deviation or as median [interquartile range] for continuous variables. Between-groups comparisons performed using chi-square for categorical variables and student’s t-test or Kruskal-Wallis nonparametric test (§) for continuous variables.

**Supplementary table 4**: bivariate comparison between controlled and uncontrolled participants, first (2009-2012) and second (2014-2017) follow-ups of the CoLaus|PsyCoLaus study, Lausanne, Switzerland. Control defined as a systolic blood pressure <130 mm Hg and a diastolic blood pressure <80 mm Hg.

|  |  | **First** |  |  | **Second** |  |
| --- | --- | --- | --- | --- | --- | --- |
|  | **Uncontrolled** | **Controlled** | **p** | **Uncontrolled** | **Controlled** | **p** |
| N | 736 | 337 |  | 764 | 393 |  |
| Age (years) | 66.0 ± 9.1 | 62.9 ± 9.4 | <0.001 | 68.6 ± 9.4 | 67.7 ± 9.6 | 0.137 |
| Women (%) | 356 (48.4) | 165 (49.0) | 0.857 | 367 (48.0) | 213 (54.2) | 0.047 |
| Swiss national (%) | 528 (71.7) | 239 (70.9) | 0.783 | 527 (69.0) | 277 (70.5) | 0.599 |
| Education (%) |  |  | 0.239 |  |  | 0.665 |
| High | 84 (11.4) | 50 (14.8) |  | 110 (14.4) | 64 (16.3) |  |
| Middle | 169 (23.0) | 80 (23.7) |  | 183 (24.0) | 89 (22.7) |  |
| Low | 483 (65.6) | 207 (61.4) |  | 471 (61.7) | 240 (61.1) |  |
| Married/couple (%) | 419 (56.9) | 181 (53.7) | 0.324 | 435 (56.9) | 212 (53.9) | 0.331 |
| Body mass index (kg/m^2^) | 28.7 ± 4.8 | 27.7 ± 5.2 | 0.004 | 28.2 ± 4.7 | 27.6 ± 5.0 | 0.029 |
| Body mass index categories (%) |  |  | 0.002 |  |  | 0.066 |
| Normal | 161 (21.9) | 102 (30.3) |  | 190 (24.9) | 122 (31.0) |  |
| Overweight | 322 (43.8) | 148 (43.9) |  | 338 (44.2) | 166 (42.2) |  |
| Obese | 253 (34.4) | 87 (25.8) |  | 236 (30.9) | 105 (26.7) |  |
| Smoking categories (%) |  |  | <0.001 |  |  | 0.117 |
| Never | 280 (38.0) | 122 (36.2) |  | 300 (39.3) | 138 (35.1) |  |
| Former | 356 (48.4) | 135 (40.1) |  | 345 (45.2) | 176 (44.8) |  |
| Current | 100 (13.6) | 80 (23.7) |  | 119 (15.6) | 79 (20.1) |  |
| Alcohol drinker (%) | 561 (76.2) | 222 (65.9) | <0.001 | 485 (70.8) | 246 (66.7) | 0.165 |
| Sedentary (%) | 430 (71.0) | 179 (69.1) | 0.586 | 313 (61.6) | 163 (62.9) | 0.722 |
| Treatment for (%) |  |  |  |  |  |  |
| Dyslipidemia | 325 (44.2) | 170 (50.5) | 0.055 | 318 (41.6) | 179 (45.6) | 0.202 |
| Diabetes | 106 (14.4) | 65 (19.3) | 0.042 | 106 (13.9) | 69 (17.6) | 0.098 |
| Number of drugs, median [IQR] |  |  |  |  |  |  |
| All, including OTC | 4 [2 - 6] | 4 [3 - 7] | <0.001 § | 4 [2 - 6] | 5 [3 - 7] | <0.001 § |
| All, excluding OTC | 3 [2 - 5] | 4 [2 - 6] | 0.001 § | 4 [2 - 6] | 4 [3 - 7] | <0.001 § |
| Antihypertensive drugs | 1 [1 - 2] | 1 [1 - 2] | 0.652 § | 1 [1 - 2] | 2 [1 - 2] | 0.061 § |
| Parental history of HT (%) | 314 (42.7) | 129 (38.3) | 0.176 | 355 (46.5) | 157 (40.0) | 0.035 |
| Genetic risk scores |  |  |  |  |  |  |
| Resistant hypertension | 6.0 ± 2.5 | 6.0 ± 2.6 | 0.946 | 5.8 ± 2.5 | 6.0 ± 2.6 | 0.303 |
| Resistant hypertension, short | 5.1 ± 2.1 | 5.0 ± 2.1 | 0.627 | 5.0 ± 2.2 | 4.9 ± 2.0 | 0.944 |
| Hypertension | 5.8 ± 2.6 | 5.9 ± 2.7 | 0.820 | 5.7 ± 2.7 | 5.7 ± 2.5 | 0.886 |

OTC, over the counter; SD, standard deviation. Results are expressed as number of participants (column %) for categorical variables and as average ± standard deviation or as median [interquartile range] for continuous variables. Between-groups comparisons performed using chi-square for categorical variables and student’s t-test or Kruskal-Wallis nonparametric test (§) for continuous variables.

**Supplementary table 5**: multivariable analysis of the associations between clinical and genetic factors with blood pressure control, first follow-up, CoLaus|PsyCoLaus study, Lausanne, Switzerland. Control defined as a systolic blood pressure <130 mm Hg and a diastolic blood pressure <80 mm Hg.

|  | **Model 1** | **p** | **Model 2** | **p** |
| --- | --- | --- | --- | --- |
| Age (per 10 years) | 0.57 (0.48 - 0.68) | <0.001 | 0.57 (0.48 - 0.69) | <0.001 |
| Men vs. women | 1.04 (0.73 - 1.48) | 0.824 | 1.05 (0.73 - 1.49) | 0.800 |
| Swiss national vs. other | 0.86 (0.61 - 1.23) | 0.414 | 0.87 (0.61 - 1.24) | 0.438 |
| Education |  |  |  |  |
| High | 1 (ref.) |  | 1 (ref.) |  |
| Middle | 0.65 (0.38 - 1.10) | 0.110 | 0.65 (0.39 - 1.11) | 0.115 |
| Low | 0.68 (0.43 - 1.09) | 0.108 | 0.68 (0.43 - 1.09) | 0.110 |
| *P-value for trend* | 0.108 |  | 0.110 |  |
| Married/couple vs. alone | 0.78 (0.56 - 1.07) | 0.126 | 0.78 (0.57 - 1.08) | 0.141 |
| Body mass index categories |  |  |  |  |
| Normal | 1 (ref.) |  | 1 (ref.) |  |
| Overweight | 0.63 (0.43 - 0.93) | 0.019 | 0.63 (0.43 - 0.93) | 0.019 |
| Obese | 0.37 (0.24 - 0.57) | <0.001 | 0.37 (0.24 - 0.58) | <0.001 |
| *P-value for trend* | <0.001 |  | <0.001 |  |
| Smoking |  |  |  |  |
| Never | 1 (ref.) |  | 1 (ref.) |  |
| Former | 0.87 (0.61 - 1.23) | 0.422 | 0.86 (0.61 - 1.23) | 0.411 |
| Current | 1.35 (0.86 - 2.11) | 0.192 | 1.34 (0.86 - 2.09) | 0.198 |
| *P-value for trend* | 0.192 |  | 0.198 |  |
| Alcohol drinker (yes vs. no) | 0.61 (0.42 - 0.87) | 0.007 | 0.60 (0.42 - 0.86) | 0.006 |
| Sedentary (yes vs. no) | 0.97 (0.68 - 1.38) | 0.870 | 0.97 (0.68 - 1.38) | 0.867 |
| Treatment for |  |  |  |  |
| Dyslipidemia (yes vs. no) | 1.16 (0.82 - 1.65) | 0.406 | 1.16 (0.81 - 1.64) | 0.418 |
| Diabetes (yes vs. no) | 1.83 (1.14 - 2.91) | 0.011 | 1.82 (1.14 - 2.90) | 0.012 |
| Number of drugs, including OTC | 1.14 (1.06 - 1.23) | 0.001 | 1.14 (1.06 - 1.23) | 0.001 |
| Parental history of HT (yes vs. no) | 0.74 (0.53 - 1.03) | 0.077 | 0.75 (0.53 - 1.04) | 0.082 |
| **Genetic risk scores** |  |  |  |  |
| Resistant hypertension, cont. | - |  | 1.02 (0.96 - 1.08) | 0.534 |
| Resistant hypertension, quartiles |  |  |  |  |
| First | 1 (ref.) |  | - |  |
| Second | 0.97 (0.63 - 1.50) | 0.883 | - |  |
| Third | 1.03 (0.66 - 1.61) | 0.885 | - |  |
| Fourth | 1.23 (0.79 - 1.90) | 0.360 | - |  |
| *P-value for trend* | 0.338 |  |  |  |

HT, hypertension; OTC, over the counter; ttt, treatment; -, not included in the model. Results are expressed as odds ratio (95% confidence interval). Statistical analyses performed using logistic regression.

**Supplementary table 6**: multivariable analysis of the associations between clinical and genetic factors with blood pressure control, second follow-up, CoLaus|PsyCoLaus study, Lausanne, Switzerland. Control defined as a systolic blood pressure <130 mm Hg and a diastolic blood pressure <80 mm Hg.

|  | **Model 1** | **p** | **Model 2** | **p** |
| --- | --- | --- | --- | --- |
| Age (per 10 years) | 0.77 (0.64 - 0.94) | 0.011 | 0.77 (0.64 - 0.94) | 0.011 |
| Men vs. women | 0.64 (0.45 - 0.91) | 0.012 | 0.63 (0.44 - 0.90) | 0.010 |
| Swiss national vs. other | 0.98 (0.69 - 1.41) | 0.932 | 1.00 (0.70 - 1.43) | 0.988 |
| Education |  |  |  |  |
| High | 1 (ref.) |  | 1 (ref.) |  |
| Middle | 0.73 (0.44 - 1.19) | 0.204 | 0.73 (0.45 - 1.19) | 0.204 |
| Low | 0.90 (0.57 - 1.40) | 0.630 | 0.90 (0.57 - 1.40) | 0.627 |
| *P-value for trend* | 0.630 |  | 0.627 |  |
| Married/couple vs. alone | 1.00 (0.71 - 1.40) | 0.997 | 0.99 (0.70 - 1.38) | 0.936 |
| Body mass index categories |  |  |  |  |
| Normal | 1 (ref.) |  | 1 (ref.) |  |
| Overweight | 0.65 (0.44 - 0.95) | 0.028 | 0.66 (0.45 - 0.96) | 0.030 |
| Obese | 0.54 (0.34 - 0.84) | 0.006 | 0.54 (0.35 - 0.85) | 0.008 |
| *P-value for trend* | 0.006 |  | 0.008 |  |
| Smoking |  |  |  |  |
| Never | 1 (ref.) |  | 1 (ref.) |  |
| Former | 0.96 (0.67 - 1.37) | 0.819 | 0.96 (0.67 - 1.37) | 0.809 |
| Current | 1.09 (0.69 - 1.74) | 0.703 | 1.11 (0.69 - 1.76) | 0.673 |
| *P-value for trend* | 0.703 |  | 0.673 |  |
| Alcohol drinker (yes vs. no) | 1.15 (0.79 - 1.67) | 0.474 | 1.16 (0.80 - 1.69) | 0.432 |
| Sedentary (yes vs. no) | 1.06 (0.75 - 1.48) | 0.744 | 1.05 (0.75 - 1.47) | 0.775 |
| Treatment for |  |  |  |  |
| Dyslipidemia (yes vs. no) | 1.04 (0.73 - 1.49) | 0.809 | 1.04 (0.73 - 1.49) | 0.809 |
| Diabetes (yes vs. no) | 1.31 (0.78 - 2.19) | 0.304 | 1.32 (0.79 - 2.19) | 0.294 |
| Number of drugs, including OTC | 1.12 (1.04 - 1.19) | 0.001 | 1.12 (1.04 - 1.19) | 0.001 |
| Parental history of HT (yes vs. no) | 0.82 (0.58 - 1.14) | 0.240 | 0.82 (0.58 - 1.14) | 0.240 |
| **Genetic risk scores** |  |  |  |  |
| Resistant hypertension, contin. | - |  | 0.99 (0.93 - 1.06) | 0.834 |
| Resistant hypertension, quart. |  |  |  |  |
| First | 1 (ref.) |  | - |  |
| Second | 1.27 (0.82 - 1.97) | 0.291 | - |  |
| Third | 1.32 (0.84 - 2.07) | 0.230 | - |  |
| Fourth | 1.09 (0.69 - 1.73) | 0.717 | - |  |
| *P-value for trend* | 0.690 |  |  |  |

HT, hypertension; OTC, over the counter; ttt, treatment; -, not included in the model. Results are expressed as odds ratio (95% confidence interval). Statistical analyses performed using logistic regression.

**Supplementary table 7:** linkage disequilibrium analysis of the SNPs included in the genetic risk score for resistant hypertension.

| **Gene** | **Variant 1** | **Variant 2** | **R^2^** | **Population** |
| --- | --- | --- | --- | --- |
| CASZ1 | rs17035646 | rs880315 | 1.000 | CEU |
| CASZ1 | rs17035646 | rs34071855 | 0.979 | CEU |
| CASZ1 | rs17035646 | rs12046278 | 0.938 | CEU |
| CASZ1 | rs34071855 | rs12046278 | 0.958 | CEU |
| CASZ1 | rs880315 | rs34071855 | 0.979 | CEU |
| CASZ1 | rs880315 | rs12046278 | 0.938 | CEU |
| EEF1DP3, FRY-AS1 | rs77270397 | rs12050053 | 0.884 | CEU |
| CASZ1 | rs17035646 | rs880315 | 0.940 | FIN |
| CASZ1 | rs17035646 | rs34071855 | 1.000 | FIN |
| CASZ1 | rs17035646 | rs12046278 | 0.843 | FIN |
| CASZ1 | rs34071855 | rs12046278 | 0.843 | FIN |
| CASZ1 | rs880315 | rs12046278 | 0.900 | FIN |
| CASZ1 | rs880315 | rs34071855 | 0.940 | FIN |
| EEF1DP3, FRY-AS1 | rs77270397 | rs12050053 | 0.568 | FIN |
| CASZ1 | rs17035646 | rs880315 | 1.000 | GBR |
| CASZ1 | rs17035646 | rs34071855 | 1.000 | GBR |
| CASZ1 | rs17035646 | rs12046278 | 0.900 | GBR |
| CASZ1 | rs34071855 | rs12046278 | 0.900 | GBR |
| CASZ1 | rs880315 | rs34071855 | 1.000 | GBR |
| CASZ1 | rs880315 | rs12046278 | 0.900 | GBR |
| EEF1DP3, FRY-AS1 | rs77270397 | rs12050053 | 1.000 | GBR |
| CASZ1 | rs17035646 | rs880315 | 1.000 | IBS |
| CASZ1 | rs17035646 | rs34071855 | 0.981 | IBS |
| CASZ1 | rs17035646 | rs12046278 | 0.941 | IBS |
| CASZ1 | rs34071855 | rs12046278 | 0.961 | IBS |
| CASZ1 | rs880315 | rs34071855 | 0.981 | IBS |
| CASZ1 | rs880315 | rs12046278 | 0.941 | IBS |
| EEF1DP3, FRY-AS1 | rs77270397 | rs12050053 | 0.707 | IBS |

CEU, Northern and Western European Ancestry in Utah, US; FIN, Finnish in Finland; GBR, British from England and Scotland; IBS, Iberian Populations in Spain. Calculations performed using 1000 genomes phase 3 data and the Linkage Disequilibrium calculator, available at <https://www.ensembl.org/Homo_sapiens/Tools/LD>.

**Supplementary table 8**: multivariable analysis of the associations between clinical and genetic factors with blood pressure control, first follow-up, CoLaus|PsyCoLaus study, Lausanne, Switzerland. Control defined as a systolic blood pressure <140 mm Hg and a diastolic blood pressure <90 mm Hg. Using the short version of the genetic risk score for resistant hypertension

|  | **Model 1** | **p** | **Model 2** | **p** |
| --- | --- | --- | --- | --- |
| Age (per 10 years) | 0.59 (0.50 - 0.70) | <0.001 | 0.60 (0.50 - 0.71) | <0.001 |
| Men vs. women | 0.86 (0.63 - 1.19) | 0.372 | 0.86 (0.63 - 1.19) | 0.369 |
| Swiss national vs. other | 1.02 (0.73 - 1.41) | 0.918 | 1.03 (0.74 - 1.42) | 0.881 |
| Education |  |  |  |  |
| High | 1 (ref.) |  | 1 (ref.) |  |
| Middle | 0.96 (0.58 - 1.58) | 0.861 | 0.96 (0.58 - 1.59) | 0.88 |
| Low | 0.96 (0.61 - 1.50) | 0.846 | 0.96 (0.61 - 1.50) | 0.856 |
| *P-value for trend* | 0.846 |  | 0.856 |  |
| Married/couple vs. alone | 0.73 (0.54 - 0.98) | 0.035 | 0.74 (0.55 - 0.99) | 0.042 |
| Body mass index categories |  |  |  |  |
| Normal | 1 (ref.) |  | 1 (ref.) |  |
| Overweight | 0.77 (0.54 - 1.10) | 0.154 | 0.77 (0.54 - 1.10) | 0.147 |
| Obese | 0.66 (0.44 - 0.98) | 0.038 | 0.66 (0.44 - 0.98) | 0.038 |
| *P-value for trend* | 0.038 |  | 0.038 |  |
| Smoking |  |  |  |  |
| Never | 1 (ref.) |  | 1 (ref.) |  |
| Former | 0.89 (0.65 - 1.22) | 0.478 | 0.89 (0.65 - 1.22) | 0.481 |
| Current | 1.31 (0.84 - 2.04) | 0.235 | 1.32 (0.85 - 2.06) | 0.219 |
| *P-value for trend* | 0.235 |  | 0.219 |  |
| Alcohol drinker (yes vs. no) | 0.74 (0.52 - 1.04) | 0.083 | 0.74 (0.53 - 1.04) | 0.086 |
| Sedentary (yes vs. no) | 0.94 (0.69 - 1.30) | 0.728 | 0.94 (0.68 - 1.29) | 0.689 |
| Treatment for |  |  |  |  |
| Dyslipidemia (yes vs. no) | 1.36 (0.99 - 1.87) | 0.059 | 1.34 (0.98 - 1.84) | 0.071 |
| Diabetes (yes vs. no) | 1.19 (0.76 - 1.86) | 0.449 | 1.18 (0.76 - 1.85) | 0.462 |
| Number of drugs, including OTC | 1.07 (1.01 - 1.15) | 0.047 | 1.07 (1.01 - 1.15) | 0.045 |
| Parental history of HT (yes vs. no) | 1.03 (0.76 - 1.39) | 0.849 | 1.04 (0.77 - 1.40) | 0.818 |
| **Genetic risk scores** |  |  |  |  |
| Resistant hypertension, cont. | - |  | 1.00 (0.93 - 1.07) | 0.981 |
| Resistant hypertension, quartiles |  |  |  |  |
| First | 1 (ref) |  | - |  |
| Second | 1.00 (0.67 - 1.48) | 0.990 | - |  |
| Third | 0.98 (0.66 - 1.45) | 0.912 | - |  |
| Fourth | 1.20 (0.81 - 1.79) | 0.363 | - |  |
| *P-value for trend* | 0.407 |  |  |  |

HT, hypertension; OTC, over the counter; ttt, treatment; -, not included in the model. Results are expressed as odds ratio (95% confidence interval). Statistical analyses performed using logistic regression.

**Supplementary table 9**: multivariable analysis of the associations between clinical and genetic factors with blood pressure control, second follow-up, CoLaus|PsyCoLaus study, Lausanne, Switzerland. Control defined as a systolic blood pressure <140 mm Hg and a diastolic blood pressure <90 mm Hg. Using the short version of the genetic risk score for resistant hypertension.

|  | **Model 1** | **p** | **Model 2** | **p** |
| --- | --- | --- | --- | --- |
| Age (per 10 years) | 0.61 (0.50 - 0.74) | <0.001 | 0.61 (0.50 - 0.75) | <0.001 |
| Men vs. women | 0.69 (0.48 - 0.97) | 0.035 | 0.68 (0.48 - 0.97) | 0.032 |
| Swiss national vs. other | 1.12 (0.78 - 1.60) | 0.542 | 1.11 (0.78 - 1.58) | 0.569 |
| Education |  |  |  |  |
| High | 1 (ref.) |  | 1 (ref.) |  |
| Middle | 0.81 (0.50 - 1.33) | 0.415 | 0.80 (0.49 - 1.32) | 0.386 |
| Low | 0.61 (0.39 - 0.96) | 0.034 | 0.61 (0.39 - 0.96) | 0.034 |
| *P-value for trend* | 0.034 |  | 0.034 |  |
| Married/couple vs. alone | 0.99 (0.71 - 1.38) | 0.941 | 1.00 (0.71 - 1.39) | 0.984 |
| Body mass index categories |  |  |  |  |
| Normal | 1 (ref.) |  | 1 (ref.) |  |
| Overweight | 0.94 (0.64 - 1.37) | 0.737 | 0.93 (0.63 - 1.36) | 0.703 |
| Obese | 1.09 (0.70 - 1.71) | 0.694 | 1.08 (0.70 - 1.69) | 0.723 |
| *P-value for trend* | 0.694 |  | 0.723 |  |
| Smoking |  |  |  |  |
| Never | 1 (ref.) |  | 1 (ref.) |  |
| Former | 0.91 (0.64 - 1.29) | 0.589 | 0.91 (0.64 - 1.30) | 0.617 |
| Current | 1.10 (0.69 - 1.77) | 0.687 | 1.07 (0.67 - 1.72) | 0.773 |
| *P-value for trend* | 0.687 |  | 0.773 |  |
| Alcohol drinker (yes vs. no) | 1.05 (0.72 - 1.52) | 0.802 | 1.04 (0.72 - 1.50) | 0.851 |
| Sedentary (yes vs. no) | 1.09 (0.78 - 1.52) | 0.612 | 1.08 (0.78 - 1.51) | 0.646 |
| Treatment for |  |  |  |  |
| Dyslipidemia (yes vs. no) | 1.39 (0.98 - 1.97) | 0.063 | 1.39 (0.98 - 1.97) | 0.063 |
| Diabetes (yes vs. no) | 0.97 (0.57 - 1.63) | 0.898 | 0.96 (0.57 - 1.63) | 0.893 |
| Number of drugs, including OTC | 1.05 (0.98 - 1.12) | 0.194 | 1.05 (0.98 - 1.12) | 0.201 |
| Parental history of HT (yes vs. no) | 0.90 (0.65 - 1.26) | 0.550 | 0.91 (0.66 - 1.27) | 0.595 |
| **Genetic risk scores** |  |  |  |  |
| Resistant hypertension, contin. | - |  | 1.02 (0.95 - 1.10) | 0.551 |
| Resistant hypertension, quart. |  |  |  |  |
| First | 1 (ref) |  | - |  |
| Second | 1.18 (0.75 - 1.84) | 0.475 | - |  |
| Third | 0.87 (0.57 - 1.33) | 0.527 | - |  |
| Fourth | 1.30 (0.85 - 2.00) | 0.230 | - |  |
| *P-value for trend* | 0.482 |  |  |  |

HT, hypertension; OTC, over the counter; ttt, treatment; -, not included in the model. Results are expressed as odds ratio (95% confidence interval). Statistical analyses performed using logistic regression.

**Supplementary table 10**: multivariable analysis of the associations between clinical and genetic factors with blood pressure control, first follow-up, CoLaus|PsyCoLaus study, Lausanne, Switzerland. Control defined as a systolic blood pressure <130 mm Hg and a diastolic blood pressure <80 mm Hg. Using the short version of the genetic risk score for resistant hypertension

|  | **Model 1** | **p** | **Model 2** | **p** |
| --- | --- | --- | --- | --- |
| Age (per 10 years) | 0.57 (0.48 - 0.69) | <0.001 | 0.58 (0.48 - 0.69) | <0.001 |
| Men vs. women | 1.05 (0.73 - 1.49) | 0.805 | 1.04 (0.73 - 1.48) | 0.834 |
| Swiss national vs. other | 0.88 (0.62 - 1.24) | 0.459 | 0.89 (0.63 - 1.26) | 0.514 |
| Education |  |  |  |  |
| High | 1 (ref.) |  | 1 (ref.) |  |
| Middle | 0.65 (0.38 - 1.10) | 0.107 | 0.66 (0.39 - 1.12) | 0.125 |
| Low | 0.67 (0.42 - 1.08) | 0.099 | 0.68 (0.43 - 1.08) | 0.105 |
| *P-value for trend* | 0.099 |  |  |  |
| Married/couple vs. alone | 0.79 (0.57 - 1.09) | 0.146 | 0.80 (0.58 - 1.10) | 0.165 |
| Body mass index categories |  |  |  |  |
| Normal | 1 (ref.) |  | 1 (ref.) |  |
| Overweight | 0.64 (0.43 - 0.93) | 0.020 | 0.64 (0.43 - 0.93) | 0.020 |
| Obese | 0.37 (0.24 - 0.58) | <0.001 | 0.38 (0.24 - 0.58) | <0.001 |
| *P-value for trend* | <0.001 |  |  |  |
| Smoking |  |  |  |  |
| Never | 1 (ref.) |  | 1 (ref.) |  |
| Former | 0.86 (0.61 - 1.23) | 0.407 | 0.86 (0.60 - 1.22) | 0.400 |
| Current | 1.34 (0.85 - 2.09) | 0.204 | 1.36 (0.87 - 2.12) | 0.18 |
| *P-value for trend* | 0.204 |  |  |  |
| Alcohol drinker (yes vs. no) | 0.60 (0.42 - 0.86) | 0.006 | 0.61 (0.43 - 0.88) | 0.007 |
| Sedentary (yes vs. no) | 0.97 (0.68 - 1.38) | 0.869 | 0.97 (0.68 - 1.37) | 0.843 |
| Treatment for |  |  |  |  |
| Dyslipidemia (yes vs. no) | 1.15 (0.81 - 1.63) | 0.428 | 1.14 (0.81 - 1.62) | 0.448 |
| Diabetes (yes vs. no) | 1.81 (1.14 - 2.90) | 0.013 | 1.81 (1.13 - 2.88) | 0.013 |
| Number of drugs, including OTC | 1.14 (1.06 - 1.23) | 0.001 | 1.14 (1.06 - 1.23) | 0.001 |
| Parental history of HT (yes vs. no) | 0.74 (0.53 - 1.04) | 0.082 | 0.75 (0.54 - 1.04) | 0.087 |
| **Genetic risk scores** |  |  |  |  |
| Resistant hypertension, cont. | - |  | 0.98 (0.91 - 1.06) | 0.622 |
| Resistant hypertension, quartiles |  |  |  |  |
| First | 1 (ref) |  | - |  |
| Second | 1.08 (0.70 - 1.67) | 0.728 | - |  |
| Third | 1.07 (0.70 - 1.65) | 0.748 | - |  |
| Fourth | 1.11 (0.72 - 1.72) | 0.631 | - |  |
| *P-value for trend* | 0.657 |  |  |  |

HT, hypertension; OTC, over the counter; ttt, treatment; -, not included in the model. Results are expressed as odds ratio (95% confidence interval). Statistical analyses performed using logistic regression.

**Supplementary table 11**: multivariable analysis of the associations between clinical and genetic factors with blood pressure control, second follow-up, CoLaus|PsyCoLaus study, Lausanne, Switzerland. Control defined as a systolic blood pressure <130 mm Hg and a diastolic blood pressure <80 mm Hg. Using the short version of the genetic risk score for resistant hypertension.

|  | **Model 1** | **p** | **Model 2** | **p** |
| --- | --- | --- | --- | --- |
| Age (per 10 years) | 0.77 (0.64 - 0.94) | 0.010 | 0.78 (0.64 - 0.94) | 0.011 |
| Men vs. women | 0.63 (0.44 - 0.90) | 0.010 | 0.62 (0.44 - 0.89) | 0.009 |
| Swiss national vs. other | 1.00 (0.69 - 1.43) | 0.982 | 1.00 (0.70 - 1.43) | 0.988 |
| Education |  |  |  |  |
| High | 1 (ref.) |  | 1 (ref.) |  |
| Middle | 0.73 (0.45 - 1.19) | 0.208 | 0.73 (0.45 - 1.20) | 0.217 |
| Low | 0.90 (0.57 - 1.40) | 0.632 | 0.90 (0.57 - 1.40) | 0.632 |
| *P-value for trend* | 0.632 |  |  |  |
| Married/couple vs. alone | 0.98 (0.70 - 1.38) | 0.923 | 0.99 (0.71 - 1.39) | 0.969 |
| Body mass index categories |  |  |  |  |
| Normal | 1 (ref.) |  | 1 (ref.) |  |
| Overweight | 0.65 (0.45 - 0.96) | 0.029 | 0.65 (0.45 - 0.96) | 0.028 |
| Obese | 0.54 (0.35 - 0.85) | 0.008 | 0.54 (0.35 - 0.85) | 0.007 |
| *P-value for trend* | 0.008 |  |  |  |
| Smoking |  |  |  |  |
| Never | 1 (ref.) |  | 1 (ref.) |  |
| Former | 0.96 (0.67 - 1.37) | 0.809 | 0.95 (0.67 - 1.37) | 0.801 |
| Current | 1.11 (0.69 - 1.76) | 0.672 | 1.11 (0.69 - 1.76) | 0.673 |
| *P-value for trend* | 0.672 |  |  |  |
| Alcohol drinker (yes vs. no) | 1.16 (0.80 - 1.69) | 0.430 | 1.16 (0.80 - 1.69) | 0.423 |
| Sedentary (yes vs. no) | 1.05 (0.75 - 1.47) | 0.772 | 1.05 (0.75 - 1.47) | 0.797 |
| Treatment for |  |  |  |  |
| Dyslipidemia (yes vs. no) | 1.04 (0.73 - 1.49) | 0.811 | 1.04 (0.73 - 1.48) | 0.821 |
| Diabetes (yes vs. no) | 1.32 (0.79 - 2.20) | 0.292 | 1.32 (0.79 - 2.20) | 0.292 |
| Number of drugs, including OTC | 1.12 (1.04 - 1.19) | 0.001 | 1.12 (1.04 - 1.19) | 0.001 |
| Parental history of HT (yes vs. no) | 0.82 (0.58 - 1.14) | 0.235 | 0.82 (0.58 - 1.14) | 0.241 |
| **Genetic risk scores** |  |  |  |  |
| Resistant hypertension, contin. | - |  | 0.97 (0.90 - 1.04) | 0.361 |
| Resistant hypertension, quart. |  |  |  |  |
| First | 1 (ref) |  | - |  |
| Second | 1.02 (0.65 - 1.59) | 0.943 | - |  |
| Third | 0.97 (0.63 - 1.49) | 0.883 | - |  |
| Fourth | 0.97 (0.63 - 1.50) | 0.899 | - |  |
| *P-value for trend* | 0.850 |  |  |  |

HT, hypertension; OTC, over the counter; ttt, treatment; -, not included in the model. Results are expressed as odds ratio (95% confidence interval). Statistical analyses performed using logistic regression.

**References**

1. Irvin MR, Sitlani CM, Floyd JS, Psaty BM, Bis JC, Wiggins KL, et al. Genome-Wide Association Study of Apparent Treatment-Resistant Hypertension in the CHARGE Consortium: The CHARGE Pharmacogenetics Working Group. Am J Hypertens. 2019;32(12):1146-53.

2. Eadon MT, Chapman AB. A Physiologic Approach to the Pharmacogenomics of Hypertension. Adv Chronic Kidney Dis. 2016;23(2):91-105.

3. Fontana V, Luizon MR, Sandrim VC. An update on the pharmacogenetics of treating hypertension. J Hum Hypertens. 2015;29(5):283-91.

4. Silva PS, Fontana V, Luizon MR, Lacchini R, Silva WA, Jr., Biagi C, et al. eNOS and BDKRB2 genotypes affect the antihypertensive responses to enalapril. Eur J Clin Pharmacol. 2013;69(2):167-77.

5. Oliveira-Paula GH, Lacchini R, Luizon MR, Fontana V, Silva PS, Biagi C, et al. Endothelial nitric oxide synthase tagSNPs influence the effects of enalapril in essential hypertension. Nitric Oxide. 2016;55-56:62-9.

6. Arwood MJ, Cavallari LH, Duarte JD. Pharmacogenomics of hypertension and heart disease. Curr Hypertens Rep. 2015;17(9):586.

7. Frau F, Zaninello R, Salvi E, Ortu MF, Braga D, Velayutham D, et al. Genome-wide association study identifies CAMKID variants involved in blood pressure response to losartan: the SOPHIA study. Pharmacogenomics. 2014;15(13):1643-52.

8. Turner ST, Bailey KR, Schwartz GL, Chapman AB, Chai HS, Boerwinkle E. Genomic association analysis identifies multiple loci influencing antihypertensive response to an angiotensin II receptor blocker. Hypertension. 2012;59(6):1204-11.

9. Hiltunen TP, Donner KM, Sarin AP, Saarela J, Ripatti S, Chapman AB, et al. Pharmacogenomics of hypertension: a genome-wide, placebo-controlled cross-over study, using four classes of antihypertensive drugs. J Am Heart Assoc. 2015;4(1):e001521.

10. Cunningham PN, Chapman AB. The future of pharmacogenetics in the treatment of hypertension. Pharmacogenomics. 2019;20(3):129-32.

11. Rimpela JM, Kontula KK, Fyhrquist F, Donner KM, Tuiskula AM, Sarin AP, et al. Replicated evidence for aminoacylase 3 and nephrin gene variations to predict antihypertensive drug responses. Pharmacogenomics. 2017;18(5):445-58.

12. Cooper-DeHoff RM, Johnson JA. Hypertension pharmacogenomics: in search of personalized treatment approaches. Nat Rev Nephrol. 2016;12(2):110-22.

13. Magvanjav O, McDonough CW, Gong Y, McClure LA, Talbert RL, Horenstein RB, et al. Pharmacogenetic Associations of beta1-Adrenergic Receptor Polymorphisms With Cardiovascular Outcomes in the SPS3 Trial (Secondary Prevention of Small Subcortical Strokes). Stroke. 2017;48(5):1337-43.

14. Si D, Wang J, Xu Y, Chen X, Zhang M, Zhou H. Association of common polymorphisms in beta1-adrenergic receptor with antihypertensive response to carvedilol. J Cardiovasc Pharmacol. 2014;64(4):306-9.

15. Chen L, Xiao T, Chen L, Xie S, Deng M, Wu D. The Association of ADRB1 and CYP2D6 Polymorphisms With Antihypertensive Effects and Analysis of Their Contribution to Hypertension Risk. Am J Med Sci. 2018;355(3):235-9.

16. Johnson JA, Zineh I, Puckett BJ, McGorray SP, Yarandi HN, Pauly DF. Beta 1-adrenergic receptor polymorphisms and antihypertensive response to metoprolol. Clin Pharmacol Ther. 2003;74(1):44-52.

17. Magvanjav O, Gong Y, McDonough CW, Chapman AB, Turner ST, Gums JG, et al. Genetic Variants Associated With Uncontrolled Blood Pressure on Thiazide Diuretic/beta-Blocker Combination Therapy in the PEAR (Pharmacogenomic Evaluation of Antihypertensive Responses) and INVEST (International Verapamil-SR Trandolapril Study) Trials. J Am Heart Assoc. 2017;6(11).

18. Singh S, El Rouby N, McDonough CW, Gong Y, Bailey KR, Boerwinkle E, et al. Genomic Association Analysis Reveals Variants Associated With Blood Pressure Response to Beta-Blockers in European Americans. Clin Transl Sci. 2019;12(5):497-504.

19. Vandell AG, Lobmeyer MT, Gawronski BE, Langaee TY, Gong Y, Gums JG, et al. G protein receptor kinase 4 polymorphisms: beta-blocker pharmacogenetics and treatment-related outcomes in hypertension. Hypertension. 2012;60(4):957-64.

20. Svensson-Farbom P, Wahlstrand B, Almgren P, Dahlberg J, Fava C, Kjeldsen S, et al. A functional variant of the NEDD4L gene is associated with beneficial treatment response with beta-blockers and diuretics in hypertensive patients. J Hypertens. 2011;29(2):388-95.

21. Gong Y, Wang Z, Beitelshees AL, McDonough CW, Langaee TY, Hall K, et al. Pharmacogenomic Genome-Wide Meta-Analysis of Blood Pressure Response to beta-Blockers in Hypertensive African Americans. Hypertension. 2016;67(3):556-63.

22. Zuo XC, Zhang WL, Yuan H, Barrett JS, Hua Y, Huang ZJ, et al. ABCB1 polymorphism and gender affect the pharmacokinetics of amlodipine in Chinese patients with essential hypertension: a population analysis. Drug Metab Pharmacokinet. 2014;29(4):305-11.

23. Beitelshees AL, Navare H, Wang D, Gong Y, Wessel J, Moss JI, et al. CACNA1C gene polymorphisms, cardiovascular disease outcomes, and treatment response. Circ Cardiovasc Genet. 2009;2(4):362-70.

24. Kamide K, Yang J, Matayoshi T, Takiuchi S, Horio T, Yoshii M, et al. Genetic polymorphisms of L-type calcium channel alpha1C and alpha1D subunit genes are associated with sensitivity to the antihypertensive effects of L-type dihydropyridine calcium-channel blockers. Circ J. 2009;73(4):732-40.

25. Bhatnagar V, Garcia EP, O'Connor DT, Brophy VH, Alcaraz J, Richard E, et al. CYP3A4 and CYP3A5 polymorphisms and blood pressure response to amlodipine among African-American men and women with early hypertensive renal disease. Am J Nephrol. 2010;31(2):95-103.

26. Langaee TY, Gong Y, Yarandi HN, Katz DA, Cooper-DeHoff RM, Pepine CJ, et al. Association of CYP3A5 polymorphisms with hypertension and antihypertensive response to verapamil. Clin Pharmacol Ther. 2007;81(3):386-91.

27. Beitelshees AL, Gong Y, Wang D, Schork NJ, Cooper-Dehoff RM, Langaee TY, et al. KCNMB1 genotype influences response to verapamil SR and adverse outcomes in the INternational VErapamil SR/Trandolapril STudy (INVEST). Pharmacogenet Genomics. 2007;17(9):719-29.

28. Sciarrone MT, Stella P, Barlassina C, Manunta P, Lanzani C, Bianchi G, et al. ACE and alpha-adducin polymorphism as markers of individual response to diuretic therapy. Hypertension. 2003;41(3):398-403.

29. Choi HD, Suh JH, Lee JY, Bae SK, Kang HE, Lee MG, et al. Effects of ACE and ADD1 gene polymorphisms on blood pressure response to hydrochlorothiazide: a meta-analysis. Int J Clin Pharmacol Ther. 2013;51(9):718-24.

30. Turner ST, Schwartz GL, Chapman AB, Boerwinkle E. C825T polymorphism of the G protein beta(3)-subunit and antihypertensive response to a thiazide diuretic. Hypertension. 2001;37(2 Pt 2):739-43.

31. McDonough CW, Burbage SE, Duarte JD, Gong Y, Langaee TY, Turner ST, et al. Association of variants in NEDD4L with blood pressure response and adverse cardiovascular outcomes in hypertensive patients treated with thiazide diuretics. J Hypertens. 2013;31(4):698-704.

32. Oliveira-Paula GH, Luizon MR, Lacchini R, Fontana V, Silva PS, Biagi C, et al. Gene-Gene Interactions Among PRKCA, NOS3 and BDKRB2 Polymorphisms Affect the Antihypertensive Effects of Enalapril. Basic Clin Pharmacol Toxicol. 2017;120(3):284-91.

33. Turner ST, Bailey KR, Fridley BL, Chapman AB, Schwartz GL, Chai HS, et al. Genomic association analysis suggests chromosome 12 locus influencing antihypertensive response to thiazide diuretic. Hypertension. 2008;52(2):359-65.
